# The Applications of Gold Nanoparticles in the Diagnosis and Treatment of Gastrointestinal Cancer

**DOI:** 10.3389/fonc.2021.819329

**Published:** 2022-01-19

**Authors:** Zhijing Yang, Dongxu Wang, Chenyu Zhang, Huimin Liu, Ming Hao, Shaoning Kan, Dianfeng Liu, Weiwei Liu

**Affiliations:** ^1^ Department of Oral and Maxillofacial Surgery, Hospital of Stomatology, Jilin University, Changchun, China; ^2^ Laboratory Animal Center, College of Animal Science, Jilin University, Changchun, China; ^3^ Jilin Provincial Key Laboratory of Tooth Development and Bone Remodeling, Hospital of Stomatology, Jilin University, Changchun, China

**Keywords:** gold nanoparticles, gastrointestinal cancer, ncRNA, imaging, cancer therapy

## Abstract

In recent years, the morbidity and mortality of gastrointestinal cancer have remained high in China. Due to the deep location of the gastrointestinal organs, such as gastric cancer, the early symptoms of cancer are not obvious. It is generally discovered at an advanced stage with distant metastasis and lymph node infiltration, making it difficult to cure. Therefore, there is a significant need for novel technologies that can effectively diagnose and treat gastrointestinal cancer, ultimately reducing its mortality. Gold nanoparticles (GNPs), a type of nanocarrier with unique optical properties and remarkable biocompatibility, have the potential to influence the fate of cancer by delivering drugs, nucleic acids to cancer cells and tissues. As a safe and reliable visualization agent, GNPs can track drugs and accurately indicate the location and boundaries of cancer, opening up new possibilities for cancer treatment. In addition, GNPs have been used in photodynamic therapy to deliver photosensitizers, as well as in combination with photothermal therapy. Therefore, GNPs can be used as a safe and effective nanomaterial in the treatment and diagnosis of gastrointestinal cancer.

## Introduction

According to the “2020 Global Cancer Report” recently released by the World Health Organization’s International Agency for Research on Cancer (IARC), the top ten new cancer cases in China in 2020 are as follows: lung, colorectal, gastric, breast, liver, esophageal, thyroid, pancreatic, prostate, and cervical cancers (WHO/IARC published the World Cancer Report 2020). Most of the cases are gastrointestinal cancers, which are closely linked to individuals with high sugar and low fiber diet, helicobacter pylori infection, sedentary, obesity, drinking, and smoking (1–5). They are generally diagnosed at an advanced stage, which seriously impacts the prognosis and life quality of patients. In order to reduce the incidence and mortality of gastrointestinal cancers, as well as improve the survival rate of patients, it is critical to excogitate the treatment and diagnosis of gastrointestinal cancers. In recent years, noble metal nanoparticles have received significant attention in cancer medical research due to their unique efficacy and specificity in imaging, diagnosis, and therapy (6–8). Gold nanoparticles (GNPs) are widely used, particularly in cancer research, because of their ease of synthesis, adjustable size and shape, remarkable biocompatibility, unique optical properties, and surface plasmon resonance (SPR) properties (9–11). Different GNPs have been designed for different types of cancers. The expression of surface receptors, and tumor environment are utilized for photothermal therapy (12), immunotherapy (13), photodynamic therapy (14), gene therapy (15), targeted therapy (16), and a combination of multiple treatments (15), allowing the integration of cancer diagnosis and treatment. This review focuses on the application of GNPs in gastrointestinal cancer.

## GNPs With Different Structures

**Figure 1:**
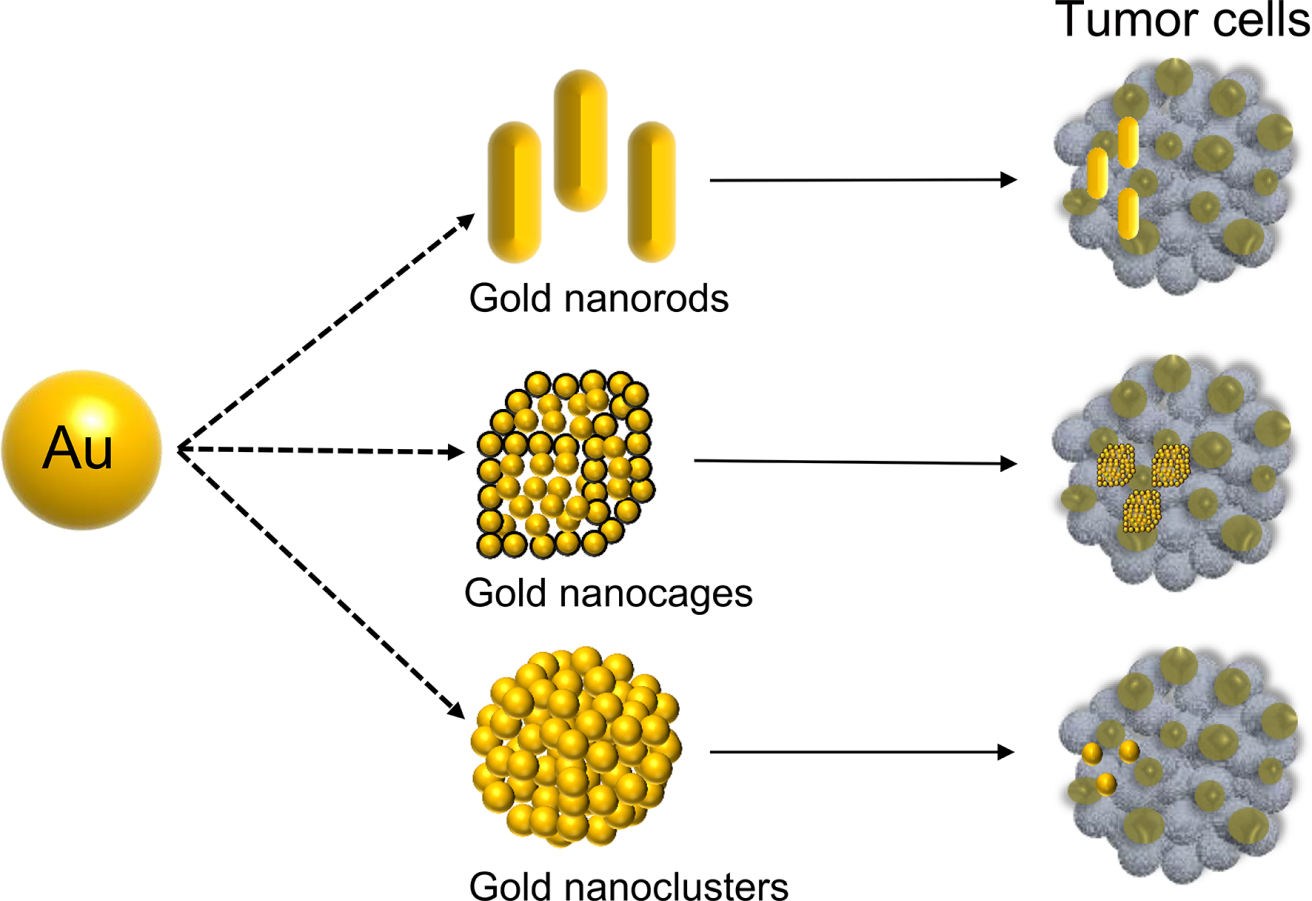
Different shapes of GNPs.

In 1857, Michael Faraday discovered the light-scattering properties of suspended gold microparticles, which is now known as the Faraday-Tyndall effect (17). Fifty years later, Hirsh et al. found that GNPs irradiated with an electromagnetic wavelength at 820 nm were able to increase the surrounding temperature, which could be used for the treatment of solid tumor (18). In July 2019, the U.S. Food and Drug Administration (FDA) approved an oral drug based on GNPs (CNM-Au8, Clene Nanomedicine, Inc.) for the treatment of amyotrophic lateral sclerosis (ALS) (19). This demonstrated that GNPs are a safe and reliable tool with great potential for disease treatment. The polarization of free electrons and the distribution of surface charges are determined by size (20, 21). GNPs are synthesized in various morphologies, shifting the absorption/scattering peak to the near-infrared window, allowing GNPs in the deep tissue to receive incident light energy (22). Over the last 20 years, many studies have reported GNPs of various shapes, including nanoclusters (23), nanorods (24), nanoplates (25), nanoshells (26), nanocages (27), and nanostars (28), which have widely studied in various cancer. In particular, gold nanorods, nanocages, and nanoclusters have been extensively used in gastrointestinal cancer (**Figure 1**).

### Gold Nanorods

Compared to the other two gold nanoparticles, the gold nanorods have short and long axis directions, with two present wavelengths, which are the plasmon resonance peaks (29). Plasmon resonance can be changed by adjusting the length-to-width ratio or effective volume of the rod (18). Therefore, it can be absorbed by the tumor tissue due to the penetration effect while maintaining a small diameter and better light absorption properties to simultaneously achieve the effect of photothermal treatment (30, 31). Furthermore, studies have shown that gold nanorods modified with low-density lipoprotein binding domain-binding polypeptide (RLT polypeptide) have a relatively evident inhibitory effect in the gastric cancer cell. In addition, they displayed significant anti-tumor efficacy in the treatment of tumors, as well as higher biosafety *in vivo* compared to the free drug doxorubicin (32). In comparison to the polymer nanomicelles F127 and Clip, a novel gold nanorod that enhances the delivery efficiency of the photodynamic drug aluminum phthalocyanine tetrasulfonic acid (AlPcS4) was developed. This gold nanorod can reduce its ability to bind serum proteins, increase the production of singlet oxygen, induce mitochondrial dysfunction, and reduce mitochondrial membrane potential to activate cell apoptosis, resulting in a highly effective anti-tumor therapy for gastric cancer (33). The EPPT-1 and the myristoylated polyarginine peptide were conjugated to the gold nanorods to target the pancreatic ductal adenocarcinoma (PDA) cells. The novel complex can induced cell death *via* plasmonic photothermal treatment (PPTT) which shows great promise for developing a new cancer therapy (34). The GNR@Mem was developed to target the oral squamous cell carcinoma (OSCC) and enhance the radiation sensitivity of OSCC cells. Moreover, combined with the photothermal therapy, it shows predominant anti-cancer effect *in vivo* (35).

### Gold Nanocages

Gold nanocages are a type of gold nanoparticles with a hollow cage-like structure. Compared to the other two gold nanoparticles, they have a high specific surface area, good surface modifiable properties, and a high drug loading rate (36, 37). Hyaluronic acid (HA), anti-GPC-1 antibody, rubescensine A, and gold nanocages were combined to inhibit pancreatic cancer. Furthermore, the multi-mode imaging ability of gold nanocages, including near-infrared fluorescence (NIRF) and magnetic resonance imaging (MRI), can detect pancreatic cancer at an early stage (38). The PDL1 antibody, TGF-β inhibitor, and gold nanocages were combined to form a complex that can selectively target colon cancer cells and accumulate in tumors. In addition to preventing primary tumor growth, the complex also inhibits distant metastasis of colorectal cancer by enhancing the distal effect mediated by synergistic immunotherapy (39). The PPHAuNCsTNCs was constructed with miR-26a loading, hyaluronic acid-modified, polyetherimide-conjugated PEGylated gold nanocages. They can accumulate in the HCC, deliver miR-26a to the tumor site and be monitored by fluorescence and photoacoustic tomography imaging (40).

### Gold Nanoclusters

Gold nanoclusters are ultra-small particles composed of several to a few hundred gold atoms (41), that have extremely low cytotoxicity and excellent red fluorescence characteristics that allow them to effectively avoid autofluorescence background *in vivo* compared to gold nanorods and nanoclusters (42). Moreover, gold nanoclusters coated with folic acid conjugated silica exhibiting excellent red fluorescence optical properties, X-ray absorption, and the ability to target gastric cancer cells, have been successfully developed. They can be used for optical and CT dual-mode imaging of gastric cancer, and have great application potential for early detection of early gastric cancer *in vivo* (43). The near-infrared fluorescent dye cy5.5 and albumin nanoparticles on the surface of gold nanoclusters can be modified to form an AuNCs/BSA-NPs complex with a significant photothermal effect. Under 808 nm laser irradiation, AuNCs/BSA-NPs can increase the temperature of colorectal cancer tumors on the surface of mice up to 50°C significantly inhibiting tumor growth, while displaying good optical fluorescence imaging properties in HCT116 tumor-bearing mice (44). The AuNCs@GSH-FA were developed with excellent biocompatibility and photostability which could target to gastric cancer to exert great excellent imaging ability for fluorescence/CT dual-mode imaging. This novel complex can acts as a promising diagnostic method for gastric cancer (45).

## GNPs as a Delivery System

**Figure 2:**
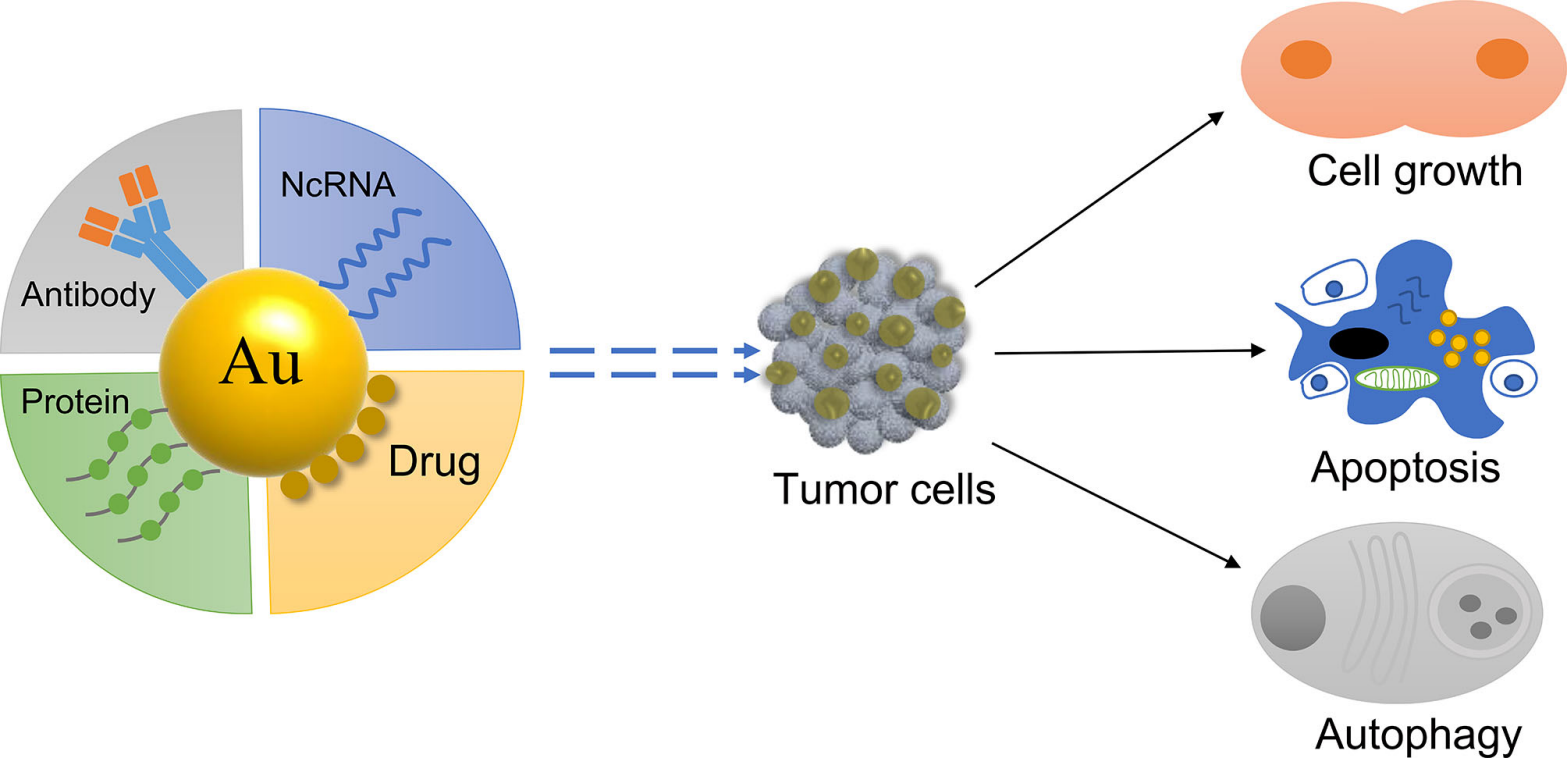
Delivery using GNPs to regulate tumor cell fate.

GNPs have a passive tumor targeting effect due to their small particle size, which can passively accumulate at the tumor site (46). Furthermore, when combined with specific active molecules, GNPs are able to actively target the tumor site and influence tumor cells (47) (**Figure 2**). For example, the ramucirumab antibody has been linked to gold nanorods to target gastric tumor and improve the anti-cancer effect of the drug (48). Therefore, gold nanomaterials can be used as a carrier to deliver chemotherapeutics and genes to the tumor site, greatly improving the efficacy of the active molecule (49, 50). The tumor microenvironment constantly changes during tumor occurrence and development (51). GNPs can reprogram the tumor microenvironment and inhibit tumor growth (52). Different types of gold nanodelivery systems have been designed to release the active molecule in the tumor site to perform an anti-tumor role depending on the conditions of the tumor microenvironment (52, 53), such as acid (54) and redox (55).

### The Role of GNPs in Drug Delivery

Although the chemotherapy drugs have anti-tumor properties, negative effects develop during the therapeutic process, such as high systemic toxicity in other organs of the body (56). Therefore, GNPs were used for targeted delivery of chemotherapy drugs to achieve precision therapy for gastrointestinal cancer (57). The targeting drug trastuzumab (Tmab) was combined with AuNCs (T-AuNPs) to create new nanocomposites that can target human epidermal growth factor receptor-2 (HER2) to induce autophagy on both Tmab sensitive and Tmab resistant gastric cancer cells (58). The anti-tumor drug epigallocatechin gallate (EGCG) is delivered by GNPs to gastric cancer cells and tissues in a time-dependent manner. This significantly inhibits proliferation compared to direct injection of the EGCG drug for gastric cancer and has no toxic effect on normal epithelial tissue (59). By encapsulating GNPs with cisplatin and glucose, cisplatin can be effectively delivered into head and neck squamous cancer (HNSCC). Compared to cisplatin used as a free drug, GNPs with cisplatin effectively inhibit tumor cell proliferation and enhance radiotherapy sensitivity in HNSCC. Furthermore, as a CT contrast agent, they can be used as an adjuvant cancer therapy in CT imaging for HNSCC diagnosis and treatment (60). Plectin-1-targeted multifunctional peptides were used to modify the GNPs to target pancreatic ductal adenocarcinoma (PDAC). Furthermore, the anti-cancer drug Gemcitabine (GEM) was conjuceted to the surface of the GNPs to form the GNPs-Gem, which can selectively deliver GEM into cancer cells and exert a significant anti-proliferative effect in PDAC cell lines (61).

### The Role of GNPs in ncRNA Delivery

MiRNAs are a type of non-coding RNAs (ncRNAs) that have a length of 20 - 22 nucleotides (62). Many studies have reported that miRNAs play an important role in the occurrence, development, and metastasis of various cancers (63). The delivery of ncRNAs to cancer cells or tissues to directly affect the expression of related proteins is an exciting prospect in cancer treatment.

However, the major limitation of using ncRNAs for cancer treatment is that they can be degraded by nuclease in serum and quickly removed *in vivo* (64, 65). Incubation of let-7a mimics with HAuCl_4_ in the tumor microenvironment leads to self-assembly of Au-let-7a NCS, which can inhibit cmyc gene expression by let-7a. This results in a significant inhibition in the migration, invasion, and proliferation of hepatocellular carcinoma (HCC) cells. Furthermore, biological imaging can be effectively carried out, and photothermal therapy can be used to induce apoptosis in HCC cells (66).

Small interfering RNAs (siRNAs) are short, double-stranded RNA that can induce mRNA degradation (67). The siRNA of nerve growth factor (NGF) was conjugated to gold nanoclusters to form GNC-siRNA, which increased the stability and prolonged the circulation time of siRNAs in blood serum. In addition, it effectively downregulates the expression of NGF and significantly inhibits tumor progression in pancreatic tumor models without significant adverse effects (68). The nanocomposites AR-GT NPs were formed by conjugating the siRNA of protein kinase B (Akt) with GNPs and encapsulating glycol chitosan taurocholic acid on the periphery, allowing them to be administered orally and effectively pass through the intestinal epithelial cells. In an orthotopic colorectal liver metastases (CLM) animal model, the nanocomposites can reduce Akt protein expression in cancer tissues and initiate tumor cell apoptosis (69).

## Application of GNPs in Cancer Diagnosis

Precise detection of tumor location and depth in patients is required for successful cancer treatment (70). Currently, imaging systems, such as computed tomography (CT) and nuclear magnetic resonance (MRI), are used for the clinical diagnosis and treatment of cancers. The GNPs are stable, nonimmunogenic and low toxicity *in vivo*. In addition, they can accumulated in the tumor sites due to the EPR effect so they are attractive in imaging diagnosis (71) (**Table 1**). Traditional CT contrast agents are small molecular iodine-based compounds with short circulation time and side effects, such as vomiting and itching, that limit their widespread use (70). GNPs are a promising CT contrast agent due to their high x-ray attenuation coefficient and biocompatibility (76, 77). A monoclonal antibody to HSP70 conjugated to GNPs was able to target mouse colon cancer cells and act as a CT contrast agent, displaying remarkable imaging ability in spectral CT and high sensitivity for the detection of even single cells (72).The Ac-PE-AuNPs was developed with favorable biocompability and remarkable X-ray attenuation property, which can accumulate in normal liver than in necrosis region caused by HCC. It can serve as a negative CT imaging agent that provide a novel diagnostic method for HCC (73). MRI is an non-invasive imaging modality that is preferred to be applied in the soft tissue imaging due to its optimal tissue contrast resolution and multiplanarity (78). The GNPs are often conjugated with T1 or T2 contrast agents to make them applied in MR imaging. For instance, the gold shell conjugated with the super paramagnetic Fe_2_O_3_ could enhance the R2 values that can get high-resolution T2*-weighted images to despict individual PANC-1 cell positions (75). The Fe3O4@Au@β-CD was developed with low r2/r1 ratio and could be a potential T2 contrast agent for MRI. Moreover, it can target to the gastric cancer cells and exhibit red fluorescence, which hold remarkable application potential diagnosis and treatment of gastric cancer (74). Furthermore, the detection sensitivity of gold nanoclusters was enhanced by utilizing the microneedles and the ultrasound to enhance the transparent efficiency, which increase the optical coherence tomography contrast level to identify the early neoplasia (79).

**Table 1:** The important uses of GNPs in the diagnosis of gastrointestinal tumors.

Particle name	Nanoparticle Size	Detection	Main results	Imaging modality	References
AuNCs@SiO2-FA	~58 nm	Gastric cancer	Targeting gastric cancer and exhibiting excellent optical property and X-ray absorbance	Fluorescence, CT imaging	(43)
CG-GNPs	20nm	Head and neck squamous cell carcinoma	Accumulating in tumor and last for 7days	CT imaging	(60)
cmHsp70-AuNPs	54 ± 11 nm	Colorectal cancer	Targeting to colorectal cancer and accumulated in tumor sites	CT imaging	(72)
PPHAuNCs-TNCs	30nm	Pancreatic ductal adenocarcinoma	Accumulated in liver and FI/PAI dual-mode imaging	PAI, Fluorescence	(40)
Ac-PE-AuNPs	95.4 ± 2.4 nm	Hepatoma carcinoma	Accumulated in normal liver than in necrosis region to serve as negative CT imaging agent	CT imaging	(73)
Fe3O4@Au@β-CD	71.40nm	Gastric cancer	Targetng to gastric cancer cells and exhibit red fluorescence,and can serve as T2 contrast agent	MRI, Fluorescence	(74)
GoldMag	~50nm	Pancreatic cancer	Serve as serve as photothermal sensitizers, and MRI is feasible to quantify delivery.	MRI	(75)

**Figure 3:**
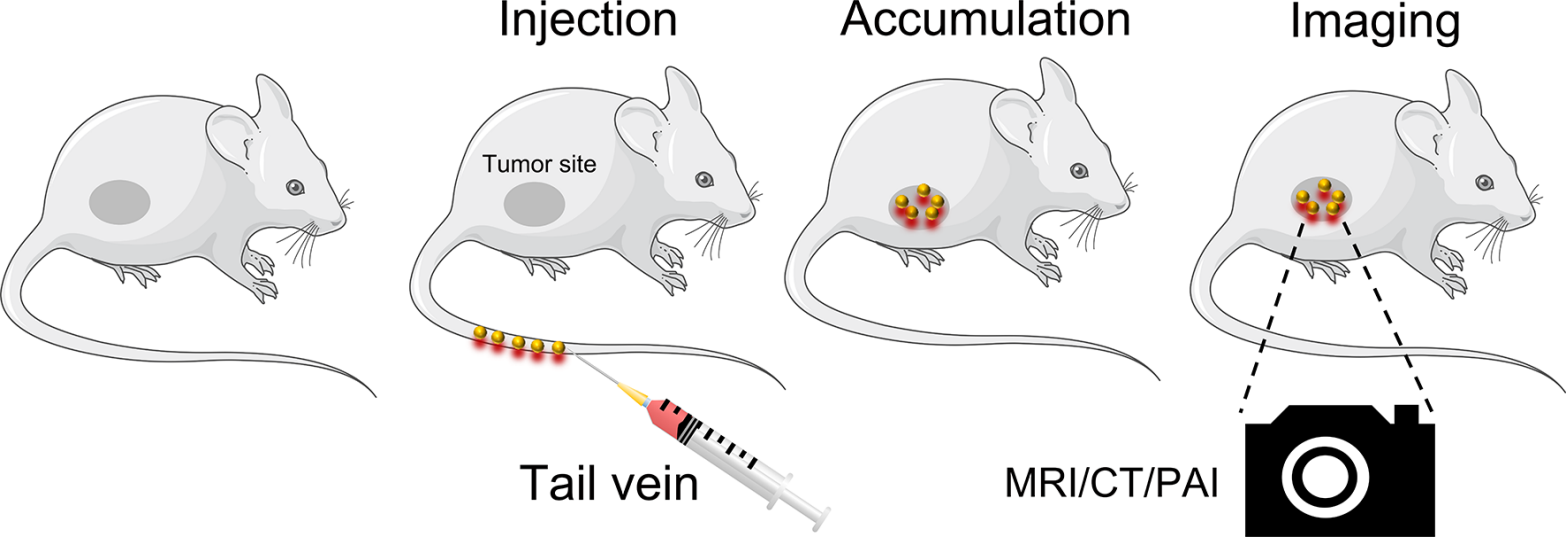
The applications of GNPs in cancer diagnosis.

Although MRI, CT are widely used in cancer diagnosis, the costs of the machines are high and the places where they can be used are limited (80). In contrast, ultrasound instruments are affordable and portable. Photoacoustic imaging (PAI) is a non-ionizing and non-invasive emerging ultrasound imaging modality that can provide high-resolution imaging in deep tissues (80–82). Compared to conventional exogenous agents, the absorption cross-section of GNCs is significantly improved due to their surface plasmon resonance (SPR) effect, which can produce strong photoacoustic signals. Furthermore, GNPs are more stable, have a higher laser damage threshold, and are biomedically inert *in vivo*, making them a promising photoacoustic imaging contrast agent (83, 84). A 5 nm molecularly activated plasmonic nanosensor (MAP) has been developed, which has better organ distribution and tissue permeability compared to larger diameter MAPs. Furthermore, it can produce a strong photoacoustic signal in the near-infrared light (NIR) region while simultaneously targeting the epidermal growth factor receptor (EGFR) to detect HNSCC with high sensitivity and specificity (85). Paclitaxel (PTX), gold nanorods, perfluorohexane (PFH), and folic acid-bovine serum protein (FA-BSA) have been combined to form the PTX-PANP-FA complex. It can act as an ultrasound contrast agent, significantly enhancing photoacoustic contrast in a mouse model. Furthermore, PTX-PANP-FA is rapidly destroyed due to PFH vaporization, resulting in quick PTX release after laser irradiation, transforming the nanocarrier into a system with drug release, imaging, and therapeutic functions (86). These reports demonstrated that GNPs act as an imaging agent that can be utilized for cancer diagnosis (**Figure 3**). Compared to conventional diagnostic contrast agents, GNPs have an additional cancer therapeutic effect.

## GNPs in Cancer Therapy

**Figure 4:**
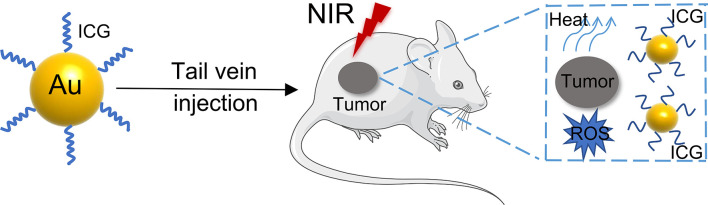
The use of PTT and PDT combination therapy to treat tumors.

At present, GNPs play an important role in the treatment of gastrointestinal cancers (**Table 2**). Photothermal therapy (PTT) is a unique cancer treatment that can selectively heat tumor tissue while avoiding damage to other tissues (89). After being irradiated by NIR, the plasmonic nanoparticles are delivered to the tumor cells or tissue, where the absorbed light is converted into heat, causing irreversible damage to the surrounding pathological tissues (90). Although a variety of nanoparticles are used for PTT, especially GNPs can passively accumulate in tumor tissue. Furthermore, their structural size can be altered to maximally absorb NIR ≈ 650 - 1350 nm) light, which has emerged as a major therapeutic platform for photothermal therapy (91). Photodynamic therapy (PDT) is a novel method for treating neoplastic diseases with photosensitizing drugs and laser activation. The photosensitizers are delivered to tumor cells or tissues and irradiated with a specific laser wavelength, producing a highly reactive oxygen species (^1^O_2_) that inhibits tumor growth (92). The most used photosensitizer is tricarbocyanine dye indocyanine green (ICG), which has been approved by the US Food and Drug Administration (FDA) as a potential near-infrared photosensitizer for clinical imaging and diagnosis (93). However, the use of ICG in fluorescence imaging and photodynamic therapy is limited by its poor stability and rapid blood clearance (94). Therefore, developing a novel cancer therapy that combines PTT and PDT to treat gastrointestinal cancer is a promising prospect (95, 96) (**Figure 4**). A PTT/PDT composite nanosystem was constructed by coupling indocyanine green (ICG) on the surface of hollow gold nanospheres (HAuNS) and subsequently modified with fal polypeptide (FAL) to target the endoplasmic reticulum (ER). When irradiated with NIR at 808 nm, there was a simultaneous increase in temperature increased and generation of ROS, inducing ER stress to enhance the immune response (87). A branched polyethylene glycol (PEI) that had a molecular weight of 10 kDa was used as a linker to conjugate ICG molecules to gold nanospheres (HAuNS). This novel nanosystem is able to inhibit tumor growth and metastasis using a combined PTT and PDT therapy mediated by NIR. Furthermore, the nanosystem is amenable to NIR fluorescence imaging, which could represent a promising approach for cancer therapy (88).

**Table 2:** The important uses of GNPs in the treatment and therapy of gastrointestinal tumors.

Particle name	Nanoparticle Size	Cell lines	Functions	Therapy and Treatment	References
GNC-Gal@CMaP	51nm	CT26	Induced immunogenic cell death and improve the anti-cancer efficiency of anti-PDL1 and TGF-β inhibitors.	PTT, Immunotherapy	(39)
AuNCs/BSA-NPs	33.8 nm	HCT116	Inhibit the cell growth and tumor growth, good optical fluorescence imaging	PTT	(44)
	~87.9 nm				
T-AuNPs	85.39 ± 0.68nm	MKN7,MKN74,NCI-N87	Inhibit the cell growth of Tmab-sensitive and Tmab-resistant gastric cancer cells through autophagy	Chemotherapy	(58)
FU-CMC-EGCG-GNPs	30~70 nm	MKN45	Inhibit the tumor growth selectively.	Chemotherapy	(59)
GNP-Gem	5.4 ± 1 nm	PANC-1, ASPC-1	Exert great anti-proliferative effect.	Chemotherapy	(61)
Au-miR-let-7a NCs	4nm	HepG2, SMMC-7721	Tumor growth inhibition,bioimaging	Gene therapy, PTT	(66)
GNC–siRNA	16.6 ± 3.0 nm	PANC-1	Knockdown the expression of NGF *in vitro* and *in vivo* to suppress tumor growth.	Gene therapy	(68)
FAL-ICG-HAuNS	151 ± 4.6 nm	CT-26	Improve the generation of ROS, inducing ER stress to enhance the immune response.	PTT, PDT, Immunotherapy	(87)
ICG-PEI-HAuNS	122.5 ± 13.5 nm	SKOV3	Reduce the cell viability, induce cell apoptosis, enhance the level of SOSG.	PTT, PDT	(88)

## 5 Gold Nanoparticles Combined With Biomolecules

**Figure 5:**
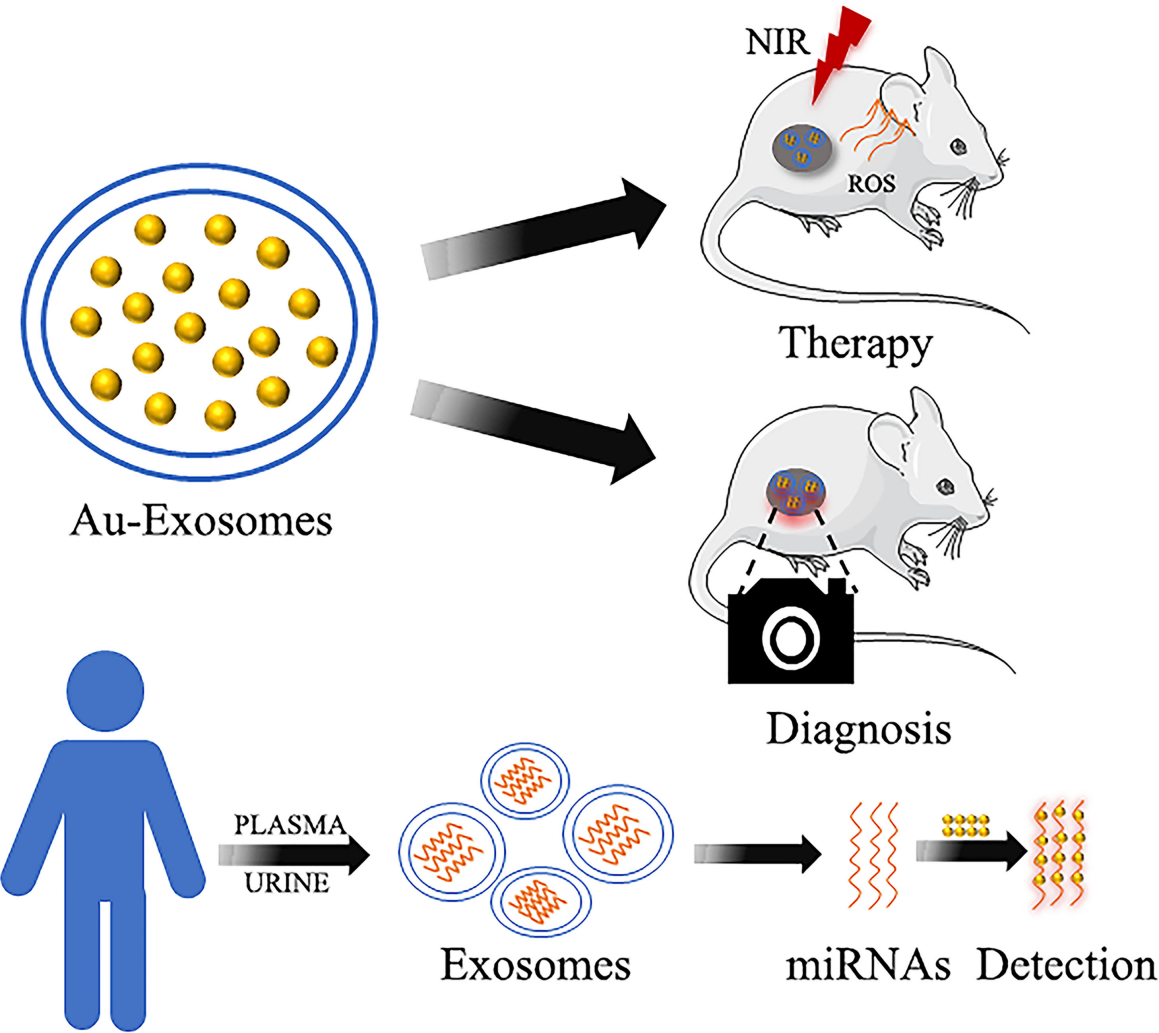
Gold nanoparticles combined with exosomes in cancer therapy and diagnosis.

Many studies have recently focused on the combination of gold nanoparticles and biomolecules to treat or diagnose tumors (97–99), especially exosomes (100, 101) (**Figure 5**). Exosomes are vesicles secreted by cells that have a diameter of 30 - 100 nm and are formed in the endocrine pathway (102, 103). They are typically composed of lipid bilayers containing membrane proteins that contain nucleic acids, such as mRNA and miRNAs, which are involved in cell communication (104–106). Due to their physical properties, such as surface plasmon resonance and scattering, GNPs can be used as a new fluorescent probe to label exosomes and analyze them using high-resolution imaging technology to trace the specific distribution of exosomes (107). Compared to traditional exosome fluorescent probes, GNPs have biocompatibility and stability advantages. Previous studies mainly focused on using exosome transport GNPs to treat cancer. For instance, GNPs were combined with proteins on the surface of exosomes, which were loaded with doxorubicin, to form the complex EVdox@AuNP. It has remarkable biocompatibility, no obvious toxicity *in vivo*, and can be used in combination with photothermal and chemotherapy for the treatment of melanoma (108). Furthermore, exosomes from the urine combined with Au-BSA@Ce6 can form a new nanocarrier EXO-PMA/Au-BSA@Ce6 that achieves real-time near-infrared fluorescence imaging and enhances PDT in gastric cancer. Compared to free Ce6, it has long-term retention, remarkable biocompatibility, superior tumor permeability, and a good targeting effect (109).

Currently, exosomes can carry the GNPs, which are used as a fluorescent imaging reagent, to specific sites in gastrointestinal cancer. The gold-iron nanoclusters were biosynthesized utilizing the tumor microenvironment by incubating HAuCl_4_, FeCl_2,_ and Na_2_SeO_3_ with tumor cells, and the tumor cells can release exosomes containing nanoparticles. These exosomes can be used as fluorescence, CT, and MRI imaging tools for the diagnosis and ablation of HCC (110). In addition, GNPs were loaded into the exosomes isolated from HNSCC cells. The exosomes were discovered to be capable of targeting and accumulating at the tumor site for CT imaging. Therefore, GNPs combined with exosomes have great potential in clinical applications (111).

Moreover, the miRNAs found in exosomes can reflect the specific physiological conditions and cellular functions of source cells (112). Detection of miRNAs can be used for early cancer diagnosis. MiRNA-21 has been targeted by developing a terahertz (THz) supramaterial biosensor for the detection of sensitive and specific exosomal miRNA in the plasma of pancreatic cancer patients. The conjugated GNPs have a high refractive index, which enhances the frequency shift of the terahertz metamaterial resonance peak. Therefore, it can be used as a promising method to detect miRNA expression levels in exosomes (113). An LSPR sensor based on complementary oligonucleotide functionalized glass substrate bonded alloy nanotubes has been developed to detect miRNA-10 in PDAC. The detection method has high sensitivity and can be used to distinguish miR-10b expression levels in PDAC, chronic pancreatitis patients, and the normal control group. This method can detect PDAC at an early stage and can be used to monitor the recurrence of PDAC after treatment or resection, which holds great clinical application promise (114).

## Discussion

**Figure 6:**
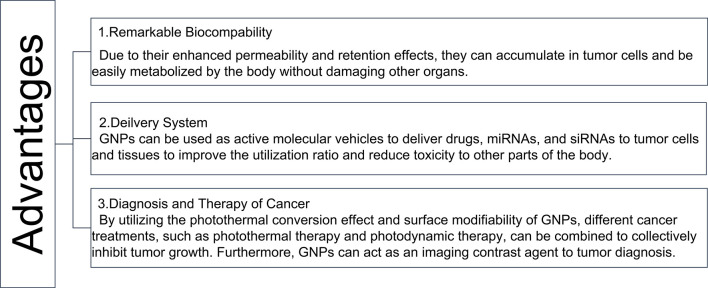
The advantages of GNPs in cancer therapy.

In recent years, the advancement of nanomedicine has given rise to new approaches for cancer treatment (115). Different shapes of GNPs and their surface modifiers have been developed according to the corresponding therapeutic effects (116). For example, gold nanomaterials modified by antibodies and target ligands have been designed to perform targeting therapy (116, 117). In addition, these GNPs can act as a delivery system, utilizing the tumor microenvironment to release and increase drug availability at the tumor site (118, 119). Furthermore, the GNCs, which have passive targeting and a highly permeable long retention effect on solid tumors, can significantly improve the therapeutic effect of drugs on tumors (120). This review mainly recapitulated the role of GNPs, which have significant diagnostic and therapeutic advantages, in gastrointestinal tumors, (**Figure 6**). GNPs have remarkable biocompatibility, allowing them to be easily taken up by cells and metabolized by the organism without causing damage to other organs (121). Furthermore, due to the photothermal conversion effect and surface modifiability of GNPs, different treatment modalities for cancer, such as PTT, PDT, immunotherapy, and chemotherapy can be combined to inhibit tumors (122, 123). The diagnosis and treatment can also be integrated through the imaging ability of GNCs (124). Furthermore, the nanodrug CYT-6091, which was created by linking human TNF alpha (rhTNF) and polyethylene glycol (PEG) to the surface of GNPs, was tested in a phase I clinical trial on a variety of solid tumors, including colon adenocarcinoma. The results showed that the highest dose of CYT-6091 outperformed the MTD of native rhTNF by 3-fold, implying that GNPs could be promising agents in clinical application (125). However, numerous challenges remain in the development process, such as drug metabolism, safety concerns, *in vivo* efficacy, biocompatibility and stability, preparation costs, and immunogenic issues. Despite the challenges that remain in the way of clinical trials, GNPs are still valuable in gastrointestinal cancer therapy and diagnosis. As a result of the extensive and successful research on GNPs in biological imaging and cancer reatment, their future clinical application is very promising to overcome the challenges of gastrointestinal cancer treatment and diagnosis.

## Conclusion

In summary, GNPs can efficiently and accurately deliver cargos, especially ncRNAs, to exhibit anti-cancer effects and be used for the diagnosis and treatment of gastrointestinal cancer. Therefore, GNPs represent a potential tool for imaging, diagnosing, and treating gastrointestinal cancers.

## Author Contributions

ZY and DW wrote the manuscript. ZY, CZ, HL, MH, SK, DL, and WL collected the references and prepared figures. All authors reviewed the manuscript. All authors contributed to the article and approved the submitted version.

## Funding

This work was supported by the Fundamental Research Funds for the Central Universities (Grant Nos. 2019JCKT-70 and 2020JCXK-45), the Jilin Province Department of Finance (Grant No. JCSZ2019378-8 and jcsz2021893-13), the Jilin Scientific and Technological Development Program (Grant Nos. 20210101010JC and 20200801077GH), and the Changchun Scientific and Technological Development Program (Grant No. 21ZY26).

## Conflict of Interest

The authors declare that the research was conducted in the absence of any commercial or financial relationships that could be construed as a potential conflict of interest.

## Publisher’s Note

All claims expressed in this article are solely those of the authors and do not necessarily represent those of their affiliated organizations, or those of the publisher, the editors and the reviewers. Any product that may be evaluated in this article, or claim that may be made by its manufacturer, is not guaranteed or endorsed by the publisher.
